# CRISPR/Cas9-targeted enrichment and long-read sequencing of the Fuchs endothelial corneal dystrophy–associated *TCF4* triplet repeat

**DOI:** 10.1038/s41436-019-0453-x

**Published:** 2019-02-08

**Authors:** Nathaniel J. Hafford-Tear, Yu-Chih Tsai, Amanda N. Sadan, Beatriz Sanchez-Pintado, Christina Zarouchlioti, Geoffrey J. Maher, Petra Liskova, Stephen J. Tuft, Alison J. Hardcastle, Tyson A. Clark, Alice E. Davidson

**Affiliations:** 1 UCL Institute of Ophthalmology, London, UK; 2 Pacific Biosciences, Menlo Park, CA USA; 3 Clinical Genetics Group, MRC Weatherall Institute of Molecular Medicine, University of Oxford, John Radcliffe Hospital, Oxford, UK; 4 Department of Ophthalmology, First Faculty of Medicine, Charles University and General University Hospital in Prague, Prague, Czech Republic; 5 Moorfields Eye Hospital, London, UK

**Keywords:** Fuchs endothelial corneal dystrophy, amplification-free sequencing, triplet repeat-mediated disease, somatic mosaicism, no-amp targeted sequencing

## Abstract

**Purpose:**

To demonstrate the utility of an amplification-free long-read sequencing method to characterize the Fuchs endothelial corneal dystrophy (FECD)-associated intronic *TCF4* triplet repeat (CTG18.1).

**Methods:**

We applied an amplification-free method, utilizing the CRISPR/Cas9 system, in combination with PacBio single-molecule real-time (SMRT) long-read sequencing, to study CTG18.1. FECD patient samples displaying a diverse range of CTG18.1 allele lengths and zygosity status (*n* = 11) were analyzed. A robust data analysis pipeline was developed to effectively filter, align, and interrogate CTG18.1-specific reads. All results were compared with conventional polymerase chain reaction (PCR)-based fragment analysis.

**Results:**

CRISPR-guided SMRT sequencing of CTG18.1 provided accurate genotyping information for all samples and phasing was possible for 18/22 alleles sequenced. Repeat length instability was observed for all expanded (≥50 repeats) phased CTG18.1 alleles analyzed. Furthermore, higher levels of repeat instability were associated with increased CTG18.1 allele length (mode length ≥91 repeats) indicating that expanded alleles behave dynamically.

**Conclusion:**

CRISPR-guided SMRT sequencing of CTG18.1 has revealed novel insights into CTG18.1 length instability. Furthermore, this study provides a framework to improve the molecular diagnostic accuracy for CTG18.1-mediated FECD, which we anticipate will become increasingly important as gene-directed therapies are developed for this common age-related and sight threatening disease.

## INTRODUCTION

Currently there are more than 40 human diseases that are caused by the expansions of simple nucleotide repeat sequences (microsatellites), with diagnosis and prognosis often dependent on accurate sizing of mutant alleles.^1^ Despite the significant advances in sequencing technologies over the past decade, microsatellites are still typically investigated in a diagnostic setting using polymerase chain reaction (PCR)-based amplification methods and fragment sizing by capillary electrophoresis. When microsatellites expand they become intractable to standard short-read next-generation sequencing (NGS) technologies due to their innate repetitive nature, size, and typically high GC content. Furthermore, when there are large differences in size between wild-type and mutant expanded alleles, as is often the case for autosomal dominant disorders, skewed allelic amplification efficiencies hinder amplification-based protocols and their analyses.^2^

Fuchs endothelial corneal dystrophy (FECD; OMIM 613267) affects up to 4.5% of individuals over 50 years of age.^3^ Approximately 75% of cases harbor a noncoding CTG microsatellite expansion termed CTG18.1, making FECD the most prevalent triplet repeat-mediated disease in humans.^4^ FECD is an age-related, degenerative condition that primarily affects the posterior corneal layers and it is the most frequent indication for corneal transplantation in the developed world.^5^ It is clinically characterized by the accelerated loss of endothelial cells and progressive thickening of Descemet membrane with focal excrescences termed guttae.^6^ In advanced disease, loss of endothelial cell function leads to corneal edema, progressive corneal opacity, and reduced vision.^7^ Expansion of CTG18.1 situated on Chr18q21.1 within an intron of *TCF4* was first shown to be significantly associated with FECD in 2012.^8^ Using a combination of short tandem repeat (STR) assays and Southern blotting the authors demonstrated that 79% of the patient cohort had at least one expanded copy of the triplet CTG motif (defined as ≥50 repeats) compared with 3% of control individuals.^8^ This striking association has subsequently been replicated in ethnically distinct populations using comparable methodologies.^4,8–10^ Typically, FECD patients harbor heterozygous expansions of the repeat in the range of 50–200 repeat units; however for a few patients, much larger expansions estimated to be up to several thousand repeat units have been identified by Southern blotting.^8,9,11^

In this study we demonstrate a custom application of an amplification-free long-read sequencing method (BioRxiv: 10.1101/203919) to specifically study the *TCF4* repeat element at a nucleotide level. The method, termed no-amp targeted sequencing, utilizes the CRISPR-Cas9 system to target, enrich, and isolate desired DNA fragments in a non–amplification dependent fashion.^12–14^ In combination with long-read single-molecule real-time (SMRT) sequencing^15,16^ this approach enabled us to analyze the disease-associated tandem repeat at a nucleotide level within a FECD patient cohort.

## MATERIALS AND METHODS

### Selection of FECD patient genomic DNA samples and STR genotyping assay

This study followed the tenets of the Declaration of Helsinki and was approved by the Moorfields Eye Hospital (MEH) ethics committee (09/H0724/25). A diagnosis of FECD was based on the presence of characteristic confluent corneal guttae on slit-lamp biomicroscopy, or a previous history of a corneal transplant for FECD. Written informed consent was obtained from all participants. Genomic DNA samples were initially genotyped for CTG18.1 using a short tandem repeat (STR) assay as described previously.^4,8^ Throughout this study expanded CTG18.1 alleles are defined as comprising ≥50 CTG repeats.

### Design of guide RNAs for Cas9 digestion

DNA sequences surrounding the CTG18.1 locus and the fragile X syndrome–associated CGG triplet repeat located within *FMR1* were used to design Cas9 guide RNAs (gRNAs). gRNAs were formed by duplexing CRISPR RNA (crRNA) with trans-activating crRNA (tracrRNA). Candidate target sequences were generated using an online CRISPR RNA configurator available on the Dharmacon website, and target specificity was checked against the human genome reference sequence (CRISPR Design Tool: https://dharmacon.horizondiscovery.com/gene-editing/crispr-cas9/crispr-design-tool/). Final crRNA sequences used for the CRISPR/Cas9 experiment were manually selected to generate a target capture region of approximately 1 kb from the Cas9 digestion site to the nearest EcoRI or BamHI digestion site, according to the hg19 reference. crRNA sequence specificity was verified by BLAST search against the human genome and the 3’ end of the crRNA was designed to be oriented toward the region of interest. crRNAs used are shown below:

*TCF4*_crRNA sequence: 5’-CAAGAGGCUAUUUACAGCUA-3’

*FMR1*_crRNA sequence: 5’-AGAGGCCGAACUGGGAUAAC-3’

### Amplification-free Cas9-targeted enrichment of the *TCF4* and *FMR1* loci

Approximately 5–20 µg of native genomic DNA was fragmented with high-fidelity restriction enzymes KpnI and EcoRV in the presence of calf intestinal alkaline phosphatase (CIP) (New England Biolabs) to first reduce genome complexity. Sample DNA was subsequently fragmented with EcoRI and BamHI (New England Biolabs) and ligated to restriction site–specific hairpin adapters carrying overhangs for EcoRI and BamHI cut sites (5’-GATCATCTCTCTCTTTTCCTCCTCCTCCGTTGTTGTTGTTGAGAGAGAT-3’ and 5’-AATTATCTCTCTCTTTTCCTCCTCCTCCGTTGTTGTTGTTGAGAGAGAT-3’) to form SMRTbell template libraries using *E. coli* DNA ligase (New England Biolabs). DNA fragments previously cut with the restriction enzymes KpnI and EcoRV were not compatible with SMRTbell adapters and were subsequently digested by exonuclease.

Next, 1 µg of SMRTbell template was digested using 32 nM Cas9 nuclease (New England Biolabs) and 48 nM target specific gRNAs (Integrated DNA Technologies) in 50 µl digestion reaction for 1 hour. crRNAs duplexed to tracrRNA at a 1:1 ratio was used for the Cas9 digestion step. Both *TCF4* and *FMR1* (positive control) gRNAs were multiplexed in the same digestion reaction. PolyA hairpin capture adapters (5’-ATCTCTCTCTTAAAAAAAAAAAAAAAAAAAAAAATTGAGAGAGAT-3’) were ligated to the DNA molecules targeted by the Cas9 digestion to form a library of asymmetrical SMRTbell template molecules.

DNA molecules, with ligated capture adapters, were enriched using a MagBead system (PacBio). Binding of capture adapters to a MagBead complex was carried out by incubating components in MagBead binding buffer for 2 hours at 4 °C. MagBeads with bound target fragments were then eluted using elution buffer (PacBio) for 10 minutes at 65 °C.

### Targeted SMRT sequencing

Target fragments were prepared for SMRT sequencing by annealing a standard PacBio primer lacking PolyA sequence and incubating at room temperature for 1 hour. Then, 0.6× AMpure Beads were used to remove unbound primer. DNA molecule/primer complexes were subsequently bound with P6 polymerase in the presence of free hairpin adapters to remove excess polymerase. Sequencing was completed using the PacBio RSII instrument with one-cell-per-well MagBead sequencing protocol, P6/C4 chemistry, and 240-minute run time. Circular consensus sequencing (CCS) reads were generated using PacBio SMRTPortal (version 2.3) software with a 90% predicted accuracy setting.

### Genome-wide coverage plots

Genome-wide coverage plots were generated for each sample from total CCS reads using PacBio SMRTPortal (version 2.3), in accordance with methods outlined previously (10.1101/203919).

### Alignment, filtering, and base calling of locus-specific CCS reads

On-target CCS reads were identified by mapping the flanking sequences on either side of the repeat region. Mapped reads were then further filtered to only retain reads with ≥90% similarity to the flanking sequences, irrespective of the CTG18.1 repeat length. On-target CCS read sequences for each sample were then visualized to identify the biallelic CTC repeat lengths, and to determine the maximum CTG18.1 repeat length present per sample. Consensus sequence mapping was also used to identify the genotype of a polymorphic intronic *TCF4* SNP situated downstream of CTG18.1 (rs599550) for each allele of each sample.

On-target CCS reads were subsequently mapped to a pool of reference sequences that included allele-specific flanking sequences (CTC repeat lengths, rs599550 genotype) and CTG repeat sizes up to the maximum length previously determined (per sample). Phasing was possible when individuals were either heterozygous for the single-nucleotide polymorphism (SNP) (rs599550) or harbored informative heterozygous CTC repeat lengths. The best matching reference sequence (≥99% match) for each CCS read was used to infer CTG18.1 repeat length. Frequency histogram plots were generated, using phasing information when possible, to show repeat length reads per sample. For samples that could not be phased, the modal repeat length per local maxima of mapped reads was identified. Mapping was completed using Blasr (https://github.com/PacificBiosciences/blasr).

*FMR1* repeat lengths were determined according the protocol previously described by Hoijer et al.^13^ using the output from a customized R script available from Github (https://github.com/NationalGenomicsInfrastructure/HTT-repeat-analysis).

## RESULTS

### Sample selection and amplification-free Cas9-targeted enrichment

To ascertain whether CRISPR-guided SMRT sequencing could effectively resolve expanded copies of the CTG18.1 locus, whole blood–derived genomic DNA samples from FECD patients with a diverse range of CTG18.1 allele lengths and zygosity status, previously defined by STR genotyping assay, were selected for analysis.^4,8^ In total, 11 DNA samples from the following categories were analyzed: 2 samples with biallelic nonexpanded (<50 repeats) alleles (category A), 5 samples with presumed monoallelic expansions (≥50 repeats) (category B), and 4 samples with presumed biallelic expansions (category C; Table 1).

**Table 1:** Results of CRISPR-guided SMRT sequencing (using 99% accuracy filtering) and short tandem repeat (STR) analysis in a Fuchs endothelial corneal dystrophy (FECD) patient cohort

Sample identifier	Category	Gender	Age at collection	Ethnicity	On-target *TCF4* reads	On-target *FMR1* reads	Phase inferred by	SNP rs599550 genotype	Allele	On-target phased *TCF4* reads	Mode *TCF4* CTG18.1 genotype		Mean *TCF4* CTG18.1 genotype		Repeat size range	Maximum repeat length	STR CTG18.1genotype	*FMR1* genotype	
											(xCTG)	(xCTC)	(xCTG)	(xCTC)				(xCGG)	(xAGG)
**Biallelic nonexpanded**																			
1	A	F	81	White British	761	204	None	TT	Allele 1	NI	11	13	NI	NI	NI	NI	12	27	2
									Allele 2		14	13					15	29	1
2	A	F	71	White British	1166	323	SNP	AT	Allele 1	445	25	13	25	13	9	30	26	28	2
									Allele 2	416	30	13	30	13	9	37	31	34	3
**Monoallelic expansion**																			
3	B	F	78	White British	970	237	SNP	AT	Allele 1	358	23	13	23	13	14	25	24	28	2
									Allele 2	375	70	13	71	13	25	90	71	32	1
4	B	M	82	White British	1011	107	CTC length and SNP	AT	Allele 1	408	23	13	24	13	59	81	24	27	2
									Allele 2	363	73	14	72	14	66	115	72	27	2
5	B	M	64	White British	1205	153	CTC length and SNP	AT	Allele 1	378	11	13	11	13	2	12	12	22	1
									Allele 2	496	80	14	82	14	98	169	79	22	1
6	B	F	65	Black African	293	115	CTC	TT	Allele 1	157	32	12	36	12	614	645	32	28	2
									Allele 2	38	110	13	171	13	466	566	109	28	2
7	B	M	42	Asian Indian	783	250	CTC	TT	Allele 1	359	17	13	17	13	2	18	18	28	2
									Allele 2	157	131	8	425	8	1244	1361	124	28	2
**Biallelic expansion**																			
8	C	F	65	White British	982	240	CTC length and SNP	AT	Allele 1	376	80	15	82	15	46	106	81	24	0
									Allele 2	367	102	11	126	11	412	498	≥81	28	2
9	C	F	85	White British	595	195	CTC	AA	Allele 1	357	72	14	74	14	170	236	72	28	0
									Allele 2	83	118	12	272	12	1524	1593	≥72	29	0
10	C	F	85	White British	1244	326	SNP	AT	Allele 1	574	69	13	70	13	35	89	70	28	2
									Allele 2	391	91	13	175	13	926	1014	≥70	37	3
11	C	M	73	White British	861	132	None	TT	Allele 1	NI	79	12	NI	NI	NI	NI	76	28	2
									Allele 2	NI	141	12					≥76	28	2

On-target reads refer to the number of Hg19 aligned reads successfully mapped to the flanking sequences on either side of each repeat region of interest (*TCF4* and *FMR1*). On-target phased *TCF4* reads refers to the number of on-target *TCF4* reads remaining after 99% accuracy filtering to a pool of phased template sequences. Repeat size range highlights the difference between the largest and smallest recorded repeat size values for a given allele.

*NI* not identifiable, *SMRT* single- molecule real-time, *SNP* single-nucleotide polymorphism.

**Fig. 1:**
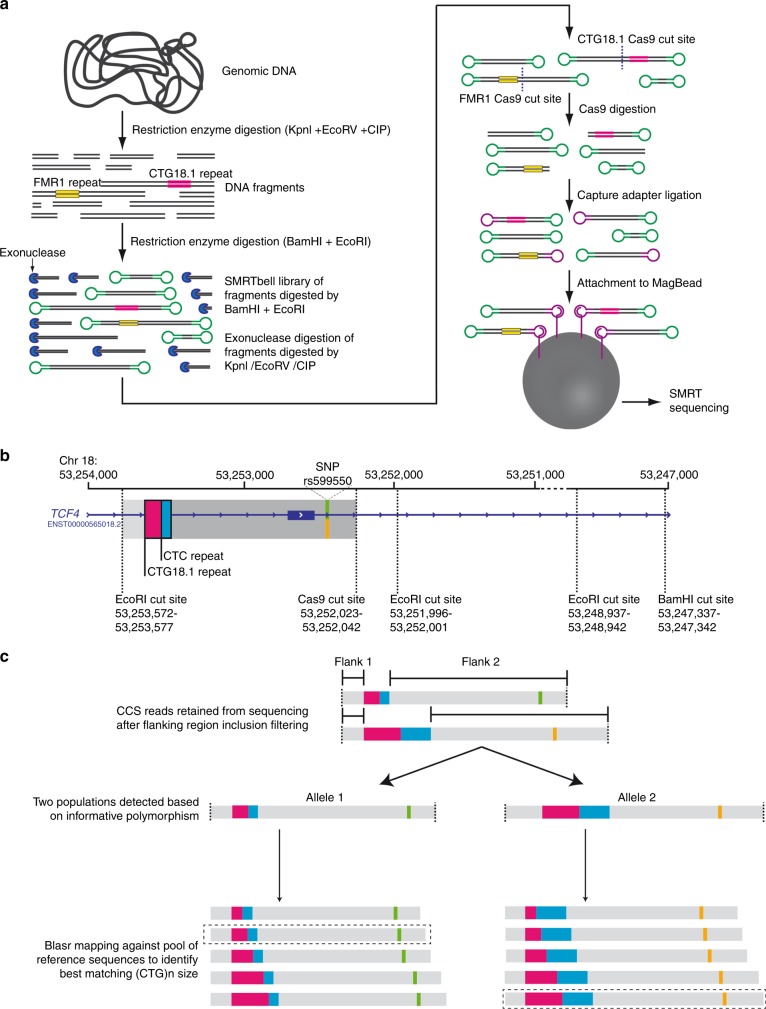
**Schematic of CRISPR-guided single-molecule real-time (SMRT) sequencing methodology, targeted capture design, and downstream analysis strategy for the CTG18.1 loci.** (**a**) First, genomic DNA underwent a complexity reduction step by digestion with selected restriction enzymes not predicted to cut inside the target region(s); nontargeted fragments were subsequently degraded by exonuclease. Targeted loci, *TCF4* CTG18.1 and *FMR1* (positive control), are depicted as pink and yellow respectively. A SMRTbell (green) library was created after target loci were excised by EcoRI and BamHI. Guide RNAs (gRNAs) targeted specifically to sequence adjacent to the desired regions (*TCF4* and *FMR1*) enabled Cas9 digestion. Cas9-digested SMRTbell fragments were ligated with engineered capture adapters (purple) and the fragments were attached to MagBeads. (**b**) EcoRI sites surrounding the CTG18.1 repeat element were identified for target capture. A gRNA Cas9 cut site was selected downstream of the CTG18.1 repeat (pink). Polymorphisms including a CTC repeat (blue) and single-nucleotide polymorphism (SNP) rs599550 (green/orange) were encompassed within the targeted region. (**c**) On-target read selection was performed by filtering reads that did not contain two flanking regions either side of the repeat locus (≥90% mapping required, not including repeat). Whenever possible CTC repeat length and/or SNP heterozygosity was used to phase circular consensus sequencing (CCS) reads. Once phased, CCS reads were mapped against a pool of reference sequences of all possible CTG18.1 repeat lengths. The reference sequence with the greatest similarity to each individual CCS read was used to infer the CTG repeat length.

An overview of the amplification-free template preparation method employed is presented in Fig. 1a. In brief, the patient-derived DNA samples underwent a series of restriction enzyme digestion steps to first reduce genome complexity, and then to introduce adapter-compatible overhangs for SMRTbell library preparation. Enrichment of the desired loci was achieved by Cas9-mediated digestions of the SMRTbell libraries using CTG18.1 (Fig. 1b) and previously optimized *FMR1*-specific^13^ gRNAs. Digested templates were enriched by ligation to new hairpin adapters and purified using MagBeads. For control purposes we used a multiplexing approach by cotargeting, enriching, and isolating a fragile X syndrome–associated CGG triplet repeat located within *FMR1*, at the same time as the FECD-associated CTG18.1 locus.

### Analysis of SMRT sequencing reads shows expected target capture

SMRTbell libraries were sequenced using a PacBio RSII instrument, generating reads of up to 7.1 kb in length.^15^ Each CCS read generated comprised multiple passes over a single DNA molecule due to the circular nature of the SMRTbell templates being sequenced.^17^ CCS reads were then mapped onto the human genome (hg19) and genome-wide coverage plots were generated (Supplementary Figure S1). Each sample-specific plot displayed clear peaks representative of on-target reads mapped to the captured loci on chromosomes 18 (CTG18.1) and X (*FMR1*). The average number of on-target CCS reads generated, per sample, was 207 for the *FMR1* loci and 897 for the *TCF4* loci (Table 1). The *FMR1* repeat region was successfully captured and analysis of the locus, as anticipated, did not detect any disease-associated repeat expansions (defined as >55 copies of the CGG repeat^18^) within our FECD cohort (Table 1). Off-target reads were observed for all samples. The most notable and consistent off-target coverage peak was observed on chromosome 21 (Chr21: 19,639,348-19,639,367). We were able to retrospectively attribute this signal to a region with similarity to the *TCF4*-specific gRNA targeted region on Chr18: 53,252,023-53,252,042. In total, four mismatches were detected between the *TCF4*-specific gRNA sequence and the nontargeted region on chromosome 21 (Supplementary Figure S2). Additionally, lower levels of off-target reads were found to consistently map to the centromeric region of chromosome 10: 42,383,760-42,393,200 (Supplementary Figure S1). However, this signal was determined to be mapping artifact due to the high levels of mismatches between the reads and reference genome (Hg19). All off-target reads generated were filtered as part of our selection and filtering strategy (see below) and hence did not affect analysis of *TCF4* or *FMR1-*specific reads.

### Selection and analysis of CTG18.1-derived SMRT sequencing reads

This is the first time, to the best of our knowledge, that CRISPR-guided SMRT sequencing has been used to generate long-read sequencing data for the CTG18.1 locus and we therefore needed to develop an analysis pipeline to effectively select and interrogate CTG18.1-specific reads. Firstly, CCS reads were filtered to exclude those that did not encompass regions flanking the repeat (Fig. 1c). Next, a preliminary analysis step was performed to estimate the maximum repeat lengths, per sample, and to determine if informative polymorphisms were present within the flanking regions. Importantly, two commonly polymorphic markers were identified within flank 2 (Fig. 1b, c): a CTC repeat located immediately 3’ to the CTG repeat of interest, and a polymorphic SNP (rs599550) 1320 bp downstream of CTG18.1. The CTC repeat lengths in our cohort ranged from 8 to 15 copies and always included one CTT repeat interruption. When individuals were found to be heterozygous for either the length of this repeat or rs599550, we had the potential to phase reads. Within this cohort we were able to phase 9/11 samples on this basis (Table 1).

**Fig. 2:**
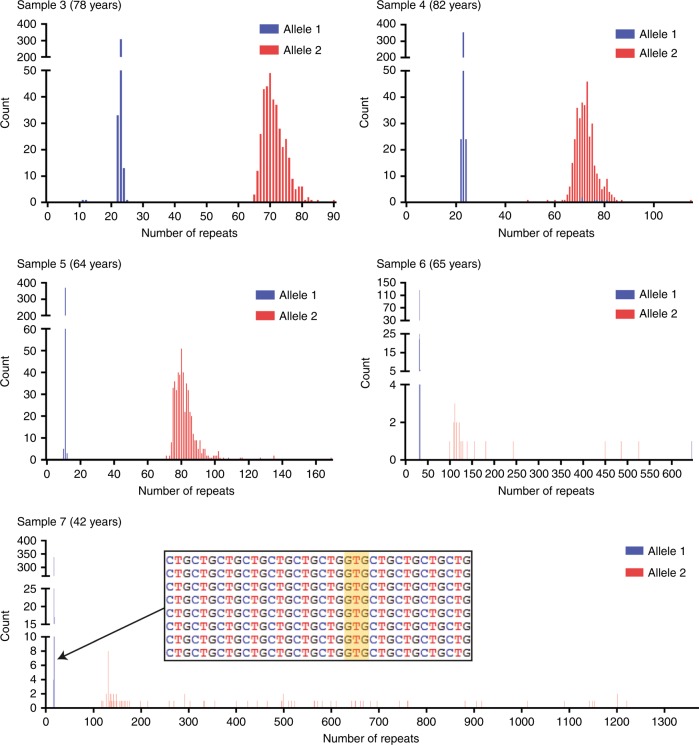
**Histograms to illustrate CTG18.1 repeat length distributions for samples harboring monoallelic expansions (category B).** Histograms show CTG18.1 repeat length read counts after filtering circular consensus sequencing (CCS) reads with ≥99% similarity to the best matched reference sequence. All samples (3–7) could be phased using CTC repeat number and/or rs599550. A single base pair interruption was identified on a single nonexpanded allele (sample 7) by overlapping and visualizing aligned CCS reads (inset).

**Fig. 3:**
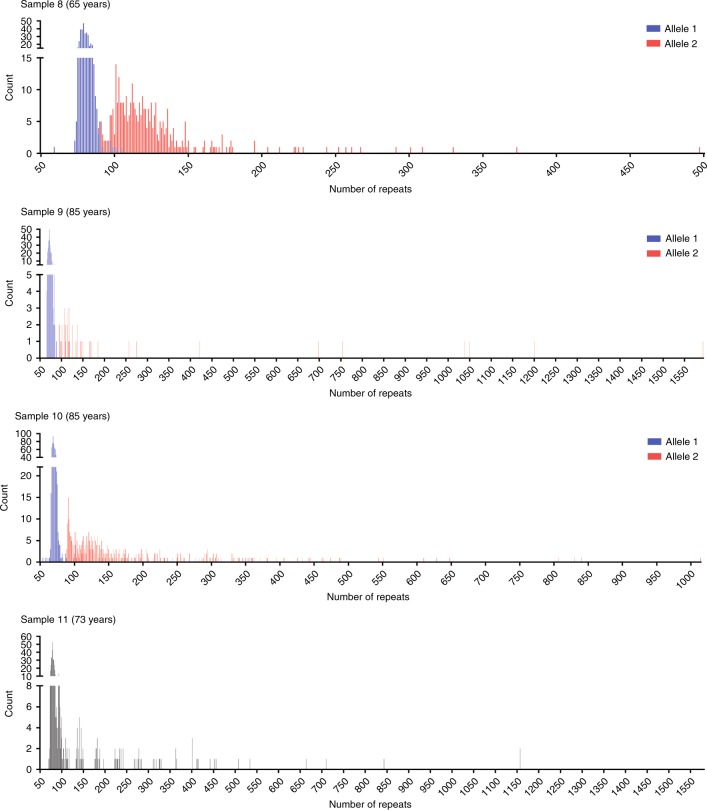
**Histograms to illustrate CTG18.1 repeat length distributions for samples harboring biallelic expansions (category C).** Histograms show CTG repeat length read counts after filtering circular consensus sequencing (CCS) reads with ≥99% similarity to the best matched reference sequence. All sequenced alleles display repeat length instability. Samples 8–10 could be phased using CTC repeat number and/or rs599550. Sample 11 was unable to be phased; however, local maxima were indicative of two alleles being detected and sequenced.

Subsequently, a more comprehensive mapping approach was employed to accurately determine repeat lengths. This involved mapping CCS reads to a customized pool of reference templates, devised after our initial *TCF4* loci–specific analysis step, which comprised all possible combinations of CTG18.1 allele lengths, in addition to the previously determined polymorphic marker genotypes, when appropriate (rs599550 and CTC repeat; Fig. 1b and c). Only reads that achieved ≥99% similarity to a sequence within the customized pool of templates were included. Sized on-target reads were then visualized and histograms were generated to depict the range of CTG18.1 repeat lengths observed in each sample (Figs. 2 and 3; Supplementary Figure S3).

### CRISPR-guided SMRT sequencing of CTG18.1 improves genotyping accuracy and reveals that expanded CTG18.1 alleles behave dynamically

Given that all samples included in this study had previously been genotyped for CTG18.1 by STR analysis we were able to make a direct comparison between the alternative methods. Average allele lengths observed by CRISPR-guided SMRT sequencing for samples comprising nonexpanded CTG18.1 alleles (categories A [*n* = 4 alleles] and B [*n* = 5 alleles]) were concordant with our previous STR analysis. However, a discrepancy of one or more repeat units per allele was noted between these differing methods. For example, sample 1 had previously been genotyped 12/15 by STR analysis whereas CRISPR-guided SMRT sequencing results suggested a genotype of 11/14 (Table 1). These differences are likely in part attributed to variability in the size of the polymorphic CTC repeat located directly 3’ of CTG18.1, given that the STR assay cannot distinguish differences in CTG18.1 length from variability in the length of flanking regions included within PCR amplimer.^4^ All sizing estimates calculated by STR analysis are based upon the reference genome (hg19), which contains 12 copies of the CTC motif. When this length deviates from 12 (e.g., sample 1), or indeed if any further insertions or deletions are present within the PCR amplified region, it will impede the accuracy of CTG18.1 sizing estimates provided by the STR assay. Data generated from category A samples (Supplementary Figure S3: Table 1) therefore exemplify that CRISPR-guided SMRT sequencing can effectively sequence biallelic nonexpanded CTG18.1 alleles and, importantly, the sequence level data generated provides improved levels of genotyping accuracy in comparison with STR analysis.

**Fig. 4:**
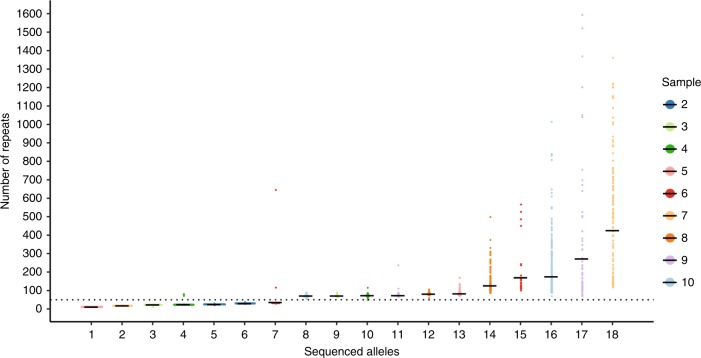
**CTG18.1 instability is correlated with repeat length.** Dot plot highlights the change in magnitude of repeat instability observed across all phased alleles (*n* = 18). Samples are arranged in order of increasing mean allele length (plotted black lines represent mean per allele). Alleles are colored in accordance to sample numbers (2–10). A dashed line represents the disease-associated threshold of 50 repeats.

Importantly, much greater differences were observed between the STR and CRISPR-guided SMRT sequencing results for all expanded alleles analyzed (categories B [*n* = 5 alleles] and C [*n* = 8 alleles]). This is attributed to the CTG18.1 instability detected for all expanded CTG18.1 alleles sequenced using the CRISPR-guided SMRT sequencing method (samples 3–11; Table 1). The histograms presented in Figs. 2 and 3 and the dot plot presented in Fig. 4 illustrate this point. For category B samples, it is apparent that alleles comprising expanded copies of CTG18.1 (≥50 copies) display greater levels of mosaicism than nonexpanded alleles (<50 copies) (samples 3–7; Fig. 2). Furthermore, levels of repeat length instability were found to increase with average allele length (Fig. 4; Table 1). For example, expanded alleles from samples 6 and 7 have mean repeat lengths of 171 and 425, repeat size ranges of 466 and 1244, and maximum repeat lengths of 566 and 1361, respectively. Whereas, samples 3, 4, and 5 all have comparatively shorter average expanded repeat lengths (71, 72, and 82), display less diverse repeat size ranges (25, 66, and 98), and maximum repeat lengths detected, per sample, are lower (90, 115, and 169) (Table 1; Fig. 2; Fig. 4). Interestingly, the same pattern is also apparent for the phased samples 8, 9, and 10; (Fig. 3) where CTG18.1 repeat length instability positively correlates with the mean CTG18.1 length (Table 1; Fig. 4).

Importantly, CRISPR-guided SMRT sequencing also enabled us to comprehensively characterize biallelic expansions of CTG18.1 for the first time. Phased samples 8, 9, and 10 all harbored biallelic expansion with average repeat lengths of 82/126, 74/272, and 70/175 respectively. Previous STR analysis of the same samples could only provide sizing estimates for the smallest alleles present in each sample (Table 1). Additionally, analysis of phased CCS reads generated from sample 7 enabled us to identify a GTG repeat interruption within the nonexpanded allele comprising 17 repeats (Fig. 2, sample 7, inset). Both these examples highlight the power of the approach and advantages of using CRISPR-guided SMRT sequencing over STR analysis to genotype CTG18.1.

### Stringency thresholds for read confidence requires compromise

Low levels of potentially incorrectly phased CCS reads were observed using our CCS read selection and analysis strategy for samples 2, 4, 6, and 9 (Figs. 2 and 3 and Supplementary Figure S3). To determine if we could improve phasing confidence, we increased the percentage similarity filtering threshold used from ≥99% to ≥99.9% and reanalyzed all samples. As described previously, sized on-target reads were then visualized and histograms were generated to depict the range of CTG18.1 repeat lengths observed in each sample. This more stringent filtering approach resulted in only one single CCS read containing an expanded copy CTG18.1 remaining phased, potentially incorrectly, to an unexpanded allele. This read was generated from sample 6 and had been phased using the CTC motif only (Table 1). However, it was noted that using this approach inappropriately filtered all reads generated from sample 7 that contain a repeat interruption, due to its absence from the reference template sequence to which the data is aligned (Fig. 2), highlighting the limitations of using such a stringent filtering threshold.

## DISCUSSION

Here we demonstrate the utility of CRISPR-guided SMRT sequencing to interrogate the FECD-associated repeat motif, CTG18.1. Our proof-of-concept study illustrates that this amplification-free targeted enrichment approach, used in combination with long-read SMRT sequencing, can generate accurate sequencing data for clinically relevant samples. Furthermore, striking levels of repeat length instability and mosaicism were observed in our studied patient cohort, highlighting that size estimates provided by conventional genotyping assays (e.g., STR and Southern blot) do not provide a robust representation of the dynamic nature of this repeat element in its expanded state. Large-scale application of CTG18.1 locus-specific CRIPSR-guided SMRT sequencing will have great diagnostic utility and will enhance our understanding of CTG18.1 genotype diversity within FECD patient and control populations in addition to furthering our understanding of phenotype–genotype correlations for this common age-related disease.

In this study, we achieved an average of 327 reads per sample and had sufficient coverage to confidently determine CTG18.1 repeat lengths for all samples analyzed. On-target read depth was however notably lower for alleles comprising larger repeats, likely attributed to the inherent size difference between molecules being sequenced. Furthermore, we were able to phase reads for 9 of 11 samples analyzed. Future adaptations of this technology could consider modifying the size of the targeted flanking regions to maximize the likelihood of encompassing informative polymorphic markers to enhance phasing capabilities. To improve coverage, future efforts should focus on (1) gRNA design to reduce off-target Cas9 activity (e.g., off -target effect observed on Chr21; Supplementary Figures S1 and S2), (2) refinement of the pull-down stage of the protocol to reduce levels of SMRTbell molecules not cleaved by Cas9 being pulled down and sequenced, and  (3) updating to the PacBio Sequel System 6.0 to enable increased multiplexing capacity at reduced cost with lower DNA input requirements. Importantly, reducing the required DNA input concentration for this method would also enable future analysis of corneal endothelial cell–derived DNA, which has the potential to provide insights into the tissue-specific nature of FECD. Furthermore, increasing multiplexing capacity will aid the diagnostic utility of the method. Notably, Tsai and colleagues have demonstrated that the method is already amenable to multiplexing across multiple genomic loci (bioRxiv: 10.1101/203919).

Instability of CTG18.1 repeats appears to consistently occur for the expanded alleles investigated in this study (*n* = 13). Furthermore, greater levels of instability are found with increased CTG18.1 length (Fig. 4) and were not found to correlate with donor age (Table 1). This phenomenon has previously been observed for other repeat expansion-mediated diseases.^13,14,19,20^ As anticipated, unexpanded alleles appeared to be relatively stable (*n* = 9). Prior to this study, all methods used to determine CTG18.1 repeat length, including STR analysis and Southern blotting, only provided crude sizing estimates and mode allele lengths.^8,21^ Our study highlights that although mode repeat lengths represent relatively reliable sizing estimates for up to approximately ≤80 copies of the repeat element, they do not provide an accurate reflection of the true distribution of allele lengths when larger unstable expansions are present. This explains the discordant mode and mean allele lengths observed for alleles comprising ≥91 copies of the repeat (Table 1). It also highlights that we should move away from considering CTG18.1 genotypes as stable entities in the expanded state and acknowledge that they are dynamic units. Future application of this technology has the potential to characterize the extent of tissue, and age-dependent mosaicism, that will likely provide both diagnostic and biological insights into CTG18.1-mediated disease mechanisms.

In this study we detected a single GTG interruption within the CTG18.1 motif on a nonexpanded allele (sample 7). This interruption was readily detectable via visualization of aligned CCS reads. Intriguingly, up to 4.2% of unaffected aged populations harbor CTG18.1 expansions but it is not yet understood why these individuals remain unaffected.^4,8^ Interruptions of disease-associated repeat expansions have been shown to modulate phenotypic expressivity by interfering with DNA and RNA stability and/or downstream gain-of-function mechanisms.^22–25^ Future application of this sequencing technology could be used to address the lack of disease observed in unaffected individuals harboring presumed CTG18.1 expansions and/or atypical phenotype–genotype correlations observed within the patient population. However, the detection of interruptions within large expanded copies of the repeat motif will likely pose a challenge given the expected prominent levels of repeat length instability predicted to occur on such alleles.

There is great clinical need to develop new FECD treatment strategies.^5,6^ Corneal transplantation is currently the only effective treatment option, and this relies upon an adequate supply of high quality donor material of which there is a global shortage.^5,26^ Given that expansion of CTG18.1 is associated with >75% of FECD cases^4,8–10^ there is now much interest in developing gene-directed, CTG18.1-mediated treatment strategies.^4,6,27^ CRISPR-guided SMRT sequencing has the potential to aid the design and implementation of CTG18.1-targeted therapies in a clinical setting, providing a diagnostic framework for accurate and high-throughput CTG18.1 genotyping and informing genotype-dependent efficacy and outcomes. Given that the disease usually presents during the fifth to sixth decade of life, CRISPR-guided SMRT sequencing also has the potential to facilitate presymptomatic detection and identify patients suitable for future preventive therapies.

In conclusion, this custom application of CRISPR-guided SMRT sequencing has provided novel insights into levels of CTG18.1 length instability within an affected FECD cohort. Furthermore, this study provides a framework for improving molecular diagnostic accuracy for FECD, which is anticipated to become increasingly important as gene-directed therapies are developed for this common age-related disease.^4,27^

## Supplementary information

Supplementary figures
