# Supplementary material for: CRISPR/Cas9-targeted enrichment and long-read sequencing of the Fuchs endothelial corneal dystrophy–associated TCF4 triplet repeat
Source: Genet Med. 2019 Feb 8;21(9):2092–102. doi: 10.1038/s41436-019-0453-x (PMC6752322; doi:10.1038/s41436-019-0453-x)
Supplement: Supplementary file 1 — Supplementary figures [file 41436_2019_453_MOESM1_ESM.docx]

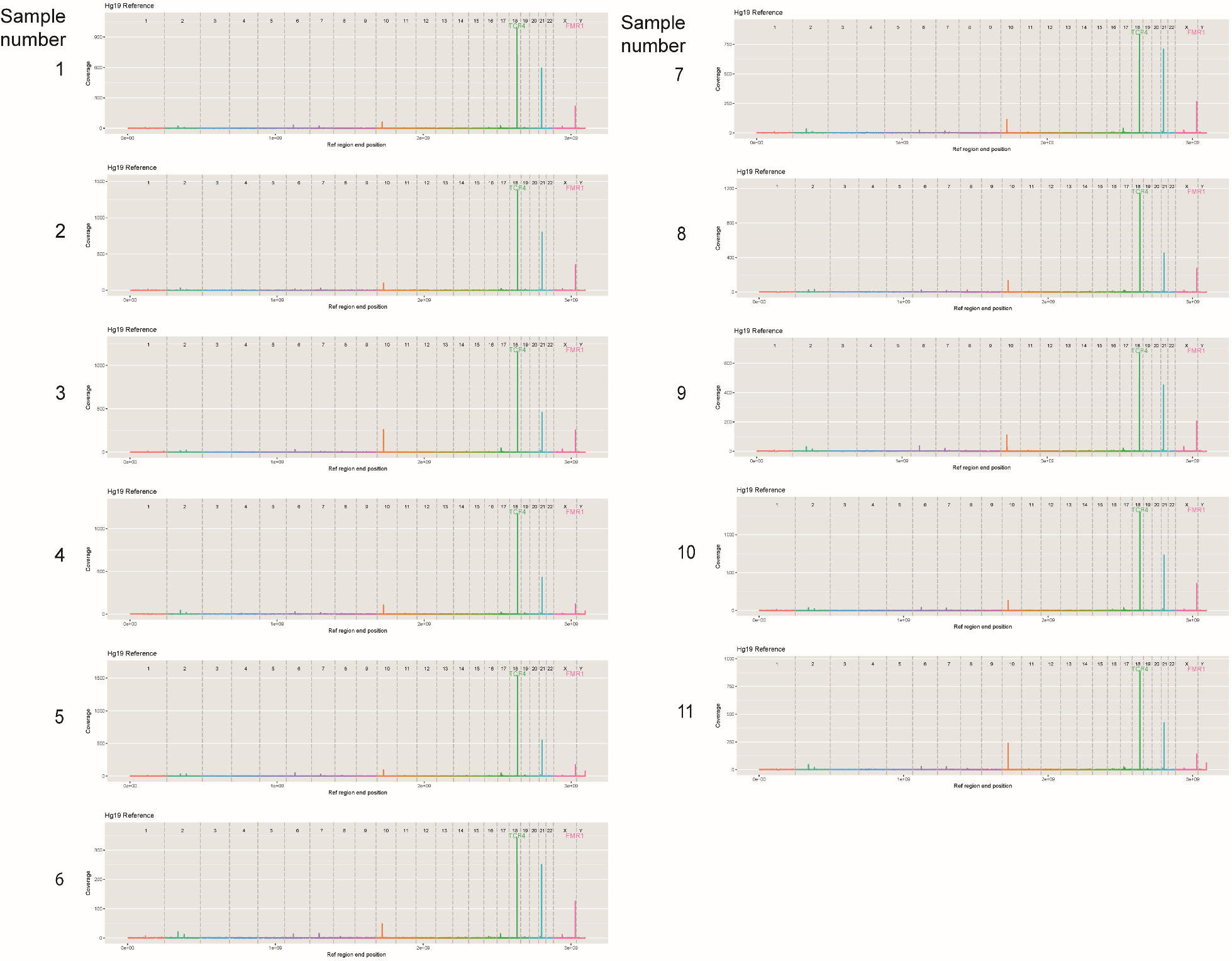
 **Figure S1: Genome wide coverage plots for samples 1-11:** Coverage plots confirmed targeted capture of the *TCF4* (chromosome 18) and *FMR1* (chromosome X) loci. The x-axes span all chromosomes within the human genome, with each distinct chromosome assigned a different colour. Consistent off-target reads were also observed across all samples (e.g. chromosomes 10 and 21).


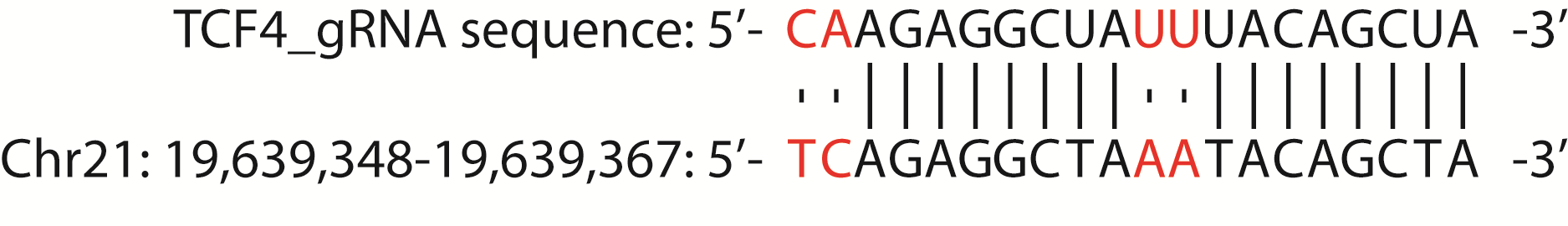


**Figure S2:** **Mis-matches determined for the off-target enrichment detected on chromosome 21**. In total, 4 mismatches were detected between the *TCF4*-specific gRNA sequence and the non-targeted region on Chromosome 21


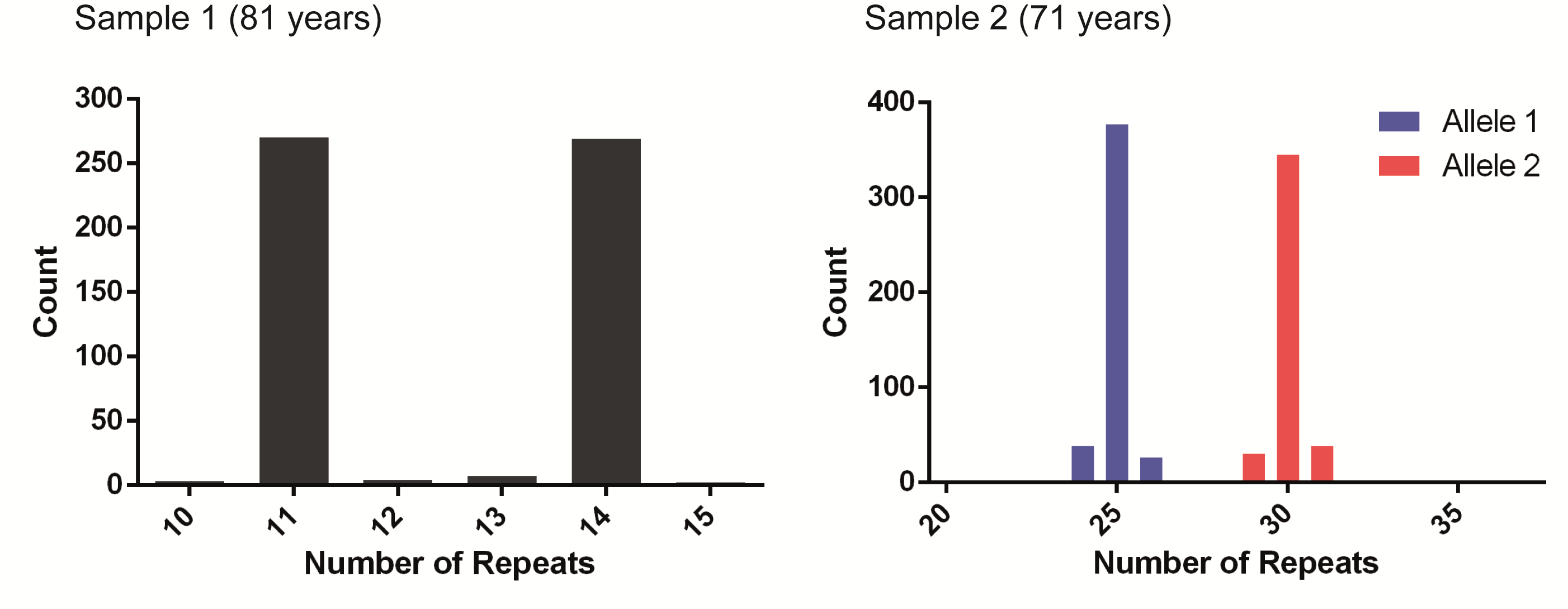


**Figure S3:** **Histograms illustrating the** **CTG18.1 repeat length distributions for samples with bi-allelic non-expanded alleles (Category A).** Histograms show CTG18.1 repeat length read counts after filtering CCS reads for ≥99 percentage similarity to the best matched reference sequence. Sample 1 was not able to be phased but showed two clear peaks, indicative of 2 alleles. Reads for sample 2 were phased using the polymorphic SNP rs599550.
